# Characterization and phylogenetic analysis of the complete mitochondrial genome in Xiaoxiang chicken (*Gallus gallus domesticus*)

**DOI:** 10.1080/23802359.2020.1715282

**Published:** 2020-01-19

**Authors:** Lili Liu, Mengya Ren, Yeqi Yang, Zhehui Chen

**Affiliations:** College of Life Science, Hunan University of Science and Technology, Xiangtan, P.R. China

**Keywords:** Mitochondrial genome, Xiaoxiang chicken, phylogenetic analysis

## Abstract

Xiaoxiang chicken (*Gallus gallus domesticus*) is one of the native breeds in the Southeastern of Guizhou province, China. The complete mitochondrial genome sequence of Xiaoxiang chicken (small-sized breed chicken) was obtained for the first time. The mitogenome is 16,784 bp in length, and it contained a D-loop region, two rRNA genes, 13 protein-coding genes, and 22 tRNA genes. A neighbour-joining phylogenetic tree was structured based on the D-loop, which indicated that the Red junglefowl was the direct ancestor of Xiaoxiang chicken, and both were closed to the Silky chicken and Dongan black chicken.

Xiaoxiang chicken (*Gallus gallus domesticus*) is the native breed in China, which is a stronger advantage in growth and breeding performance, and got the ability to tolerate or resist disease. It has the physical characteristics of small-sized, yellow-brown flat feathers and black-colored bones. Moreover, the meat of Xiaoxiang chicken is tender and healthy food with the nutrition value of much protein and lower in fat, and better medicine function in Chinese indigenous chickens (Li et al. 2012). The adult individuals of Xiaoxiang chicken were collected from Rongjiang County (E108°04′–108°44′, N25°26′–26°28′), the Southeastern of Guizhou province, China. Total DNA was extracted from the specimens (Voucher No. XXC160802) were stored at −70 °C in the Laboratory of Molecular Biology, College of Life Science, Hunan University of Science and Technology. We reported the sequence of the complete mitogenome of Xiaoxiang chicken for the first time, and the complete mitochondrial DNA data from this study have submitted to GenBank and got the accession number (KX781319). Based on the mitochondrial genome of Silky chicken (AB086102.1) to design about 22 pairs of primers for amplifying the complete mitochondrial DNA, and the polymerase chain reaction (PCR) products of the Gel electrophoresis were purified using Gel AdvancedTM Gel Extraction (Rich Biotech, China) and sequenced using BioSune Biotech (Shanghai, China). The characters of base composition and distribution were analyzed using tRNA Scan-SE1.21 and DOGMA software (Liu et al. 2016; Yu et al. 2016). The mitochondrial DNA sequence was analyzed using the DNAStar7.1 software (Madison, WI). Based on D-loop sequence of Xiaoxiang chicken, the phylogenetic tree was constructed using MEGA5.05 and N-J Algorith software according to previous methods (Liu et al. 2018).

The results revealed that the total length of mitochondrial sequence is 16,784 bp, with the base composition of 30.3% for A, 23.8% for T, 32.4% for C, 13.5% for G in the Xiaoxiang chicken. The length of non-coding region is 1231 bp, which accounts for 7.33% of the total length, and as the D-loop region. The length of coding region is 15,553 bp, and it contained 37 coding genes (13 protein-coding genes, two rRNA genes, and 22 tRNA genes), 48 bp intergenic spacer, and 31 bp overlap regions. 22 tRNA genes were assigned between rRNA and protein-coding genes, ranging from 65 to 76 bp. One protein-coding gene (ND6) and eight tRNA genes were encoded on the light (L) strand including tRNA^Cys^, tRNA^Tyr^, tRNA^Ser^, tRNA^Pro^, tRNA^Glu^, tRNA^Gln^, tRNA^Ala^, and tRNA^Asn^. However, the other 12 protein-coding genes, 14 tRNA, and two rRNA genes were located in heavy(H) strand. The initiation codon of proteins genes was ATG except for COX1 being GTG. There are four types of termination codon for proteins genes, including AGG, TAG, TAA, and an incomplete termination codon “T––”, which is the 5’ terminal of adjacent gene (Anderson et al. 1981). Xiaoxiang chicken was similar to other vertebrates. The lengths of 12s rRNA and16s rRNA were 976 bp and 1622 bp, which located between the tRNA^Leu^ and tRNA^Phe^ genes and separated by the tRNA^Val^ gene.

The maximum-likelihood phylogenetic tree was constructed according to D-loop sequence of 11 chicken breeds (Figure 1). We assumed that Xiaoxiang black-boned chicken has the closest relationship with the Silky and grouped to one class with Dongan black chicken, *Gallus gallus*, Taoyuan chicken, Guangxi three-buff chicken, and White plymouth rock. However, Xiaoxiang chicken is the fastest distance with the *Coturnix chinensis*. This work provides molecular basis for the study of origin, phylogeny, and evolution of *Gallus gallus domesticus*.

**Figure 1.**
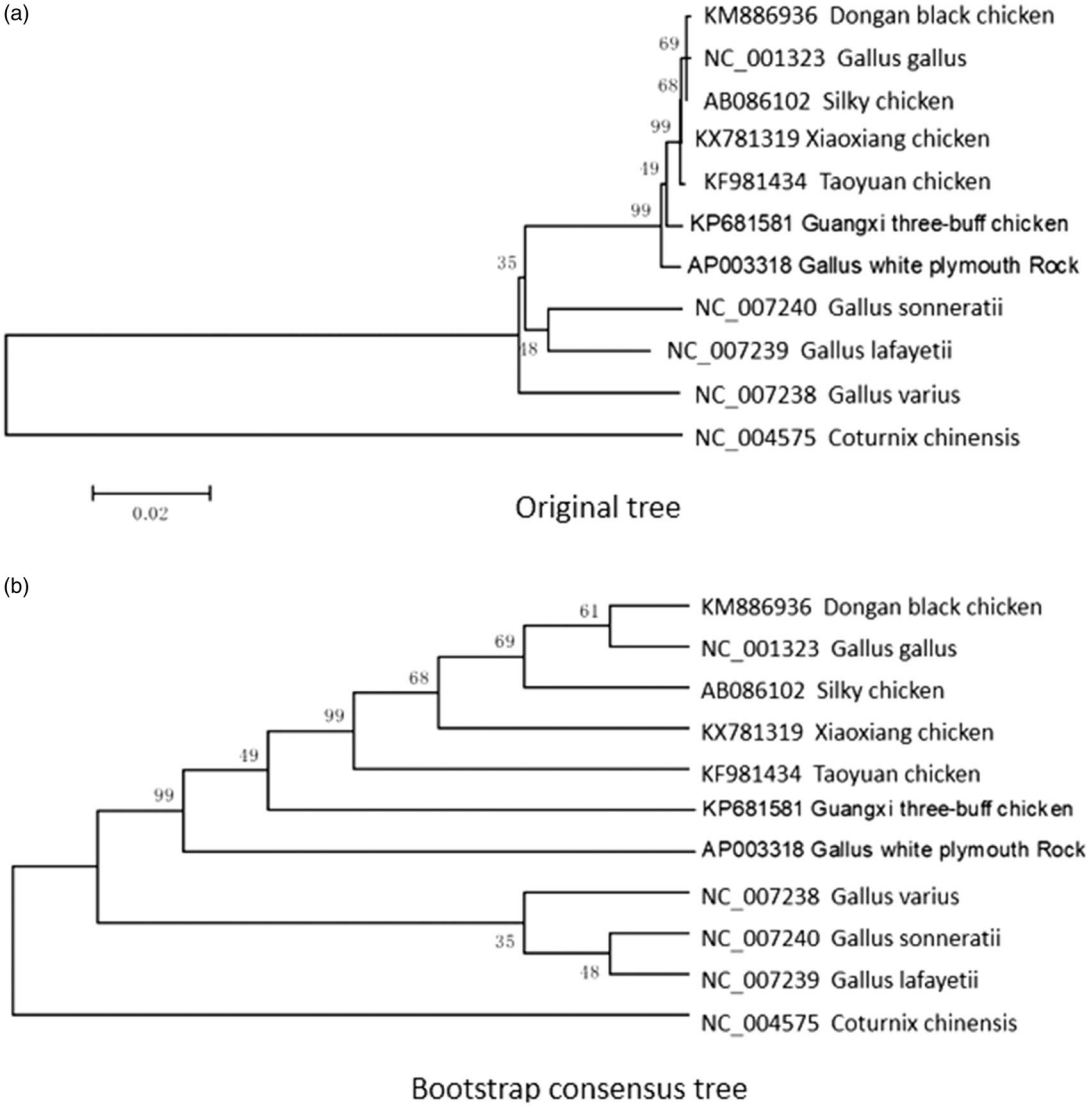
Based on D-loop sequence to construct phylogenetic tree (a. Original tree, b. Bootstrap consensus tree) in 11 chicken breeds. The mitochondrial DNA sequences are downloaded from GenBank, and the phylogenetic tree is constructed using a maximum-likelihood method on MEGA 5.05. The gene’s accession number for tree construction is listed as follow, Dongan black chicken (KM886936); *Gallus gallus* (NC_001323); Silky chicken (AB086102); Xiaoxiang chicken (KX781319); Taoyuan chicken (KF981434); Guangxi three-buff chicken (KP681581); *Gallus* white plymouth Rock (AP003318); *Gallus sonneratii* (NC_007240); *Gallus lafayettei* (NC_007239); *Gallus varius* (NC_007238); *Coturnix chinensis* (NC_004575).
